# Pain-related presentations to a general pediatric outpatient clinic and physicians’ approaches

**DOI:** 10.3389/fped.2026.1923841

**Published:** 2026-10-07

**Authors:** Zeynep Büşra Akgün, Fatma Zehra Öztek Çelebi, M. Melek Oğuz, Esma Altınel Açoğlu, Meltem İleri, Hüsniye Yücel

**Affiliations:** 1 Department of Pediatrics, Ankara Etlik City Hospital, Ankara, Türkiye; 2 University of Health Sciences, Ankara Etlik City Hospital, Ankara, Turkey

**Keywords:** acute pain, chronic pain, pain epidemiology, pain managemant, pediatric pain

## Abstract

**Background:**

Pain is a common reason for pediatric outpatient visits, yet deficiencies in assessment and management remain widespread. This study aimed to determine the proportion of pediatric outpatient visits involving pain, classify pain as acute or chronic, evaluate associations with age and sex, assess physicians’ diagnostic approaches, and examine pain management and follow-up.

**Methods:**

This ambispective observational study combined a retrospective cross-sectional assessment of pain-related outpatient visits with a prospective follow-up of patients with chronic pain. Children aged 3–18 years attending Ankara Etlik City Hospital between February 1 and 28, 2023, were included. Demographic and pain-related data, clinical findings, laboratory results, diagnoses, and treatments were retrospectively extracted from the hospital information system using a standardized data collection form. Patients with chronic pain were prospectively followed for 6 months; follow-up attendance was assessed through the hospital information system, and those who missed scheduled visits were contacted by telephone. Multivariable logistic regression analyses were performed to identify factors independently associated with chronic pain and analgesic prescribing.

**Results:**

During the study period, 646 of 6,790 outpatient visits (9.5%) involved pain-related complaints; 53.6% of patients were female. Acute pain was reported in 70.1%, chronic pain in 10.8%, and pain duration was undocumented in 19.0%. Adolescents accounted for 64.3% of chronic pain cases (*p* = 0.001). Each one-year increase in age was associated with an 11% increase in the odds of chronic pain (aOR 1.11, 95% CI 1.05–1.18), and female sex was associated with 1.85-fold higher odds of chronic pain (aOR 1.85, 95% CI 1.07–3.19). Diagnostic testing was performed in 58.2% of patients, and 38.5% of those tested had normal results. Only 26.0% received analgesic treatment. Abnormal physical examination findings were the only independent predictor of analgesic prescribing (aOR 5.10, 95% CI 3.25–7.98; *p* < 0.001). Among 70 patients with chronic pain, follow-up information was available for 45; 20 (44.4%) reported persistent pain, 11 (24.4%) improvement, and 14 (31.1%) complete resolution.

**Conclusion:**

Standardized pain assessment, structured documentation, and evidence-based management may improve pediatric pain care.

## 1 Introduction

Pain is a common health problem in children and adolescents and may occur in both acute and chronic forms. Previous epidemiological studies have reported considerable variation in the frequency of pediatric pain, depending on the population studied, the definition of pain, the assessment method, and the duration considered. Recurrent or chronic pain has been estimated to affect approximately 15%–25% of children and adolescents, with headache, abdominal pain, and musculoskeletal pain among the most frequently reported pain conditions (1). Community-based studies have similarly demonstrated wide variation in reported pain frequencies; headache prevalence has ranged from 23% to 51%, whereas abdominal pain prevalence has ranged from 10% to 27% (2). These differences highlight the methodological heterogeneity of existing epidemiological studies and limit direct comparisons across populations.

Although pediatric pain has been extensively investigated in community- and school-based populations, less is known about how pain presents and is managed in routine general pediatric outpatient practice. In particular, real-world data simultaneously describing the proportion of outpatient visits involving pain, the distribution of acute and chronic pain, associated clinical characteristics, diagnostic evaluation, analgesic prescribing, and subsequent follow-up remain limited (3). This distinction is clinically important because the characteristics of children presenting to healthcare services cannot be directly inferred from population-based prevalence estimates.

Assessment and management of pediatric pain remain challenging in routine clinical practice. Accurate and developmentally appropriate pain assessment is considered an essential component of effective pain management. Inadequate recognition or documentation of pain may affect subsequent diagnostic evaluation and treatment, while the absence of standardized assessment practices may contribute to variability in clinical management (4, 5). Characterizing current practice in general pediatric outpatient settings may therefore help identify potential gaps in pain assessment, documentation, treatment, and follow-up and may inform strategies to improve pediatric pain care.

Accordingly, the present study aimed to determine the proportion of general pediatric outpatient visits involving a pain complaint; describe the distribution of acute and chronic pain according to age and sex; characterize pain location, associated clinical findings, and diagnostic evaluation; assess analgesic prescribing; and evaluate the follow-up of patients with chronic pain.

## 2 Methods

### 2.1 Study design and setting

This ambispective observational study consisted of a retrospective cross-sectional review of pediatric outpatient visits and a prospective follow-up of patients with chronic pain. The reporting of the Methods section was guided by the STROBE (Strengthening the Reporting of Observational Studies in Epidemiology) recommendations. The study was conducted in the Department of Pediatrics at Ankara Etlik City Hospital and included patients aged 3–18 years who presented to the general pediatric outpatient clinic during February 2023. All consecutive eligible outpatient visits during the predefined study period were screened for inclusion.

### 2.2 Study population and eligibility criteria

During the predefined study period, 6,790 outpatient visits by patients aged 3–18 years were recorded in the general pediatric outpatient clinic. All eligible outpatient visits during this period were screened for the presence of a pain complaint. Visits involving pain were identified through a systematic review of the presenting complaint and medical history documented in the anamnesis notes in the hospital information system. Records containing the term ‘pain’ were screened, and identified records were subsequently reviewed in detail to confirm the presence, location, and duration of the pain complaint. A total of 646 visits involving a pain complaint were identified and included in the detailed evaluation. No formal sample size calculation was performed because all eligible outpatient visits during the predefined study period were screened for inclusion.

Patients younger than 3 years were excluded because reliable self-reporting of pain was considered difficult in this age group. Patients who were unable to communicate pain because of neurological, developmental, or other conditions affecting verbal communication were also excluded.

### 2.3 Data collection and study variables

Clinical and demographic data were obtained from the hospital information system and extracted using a predefined, standardized data collection form. The proportion of all eligible pediatric outpatient visits involving a pain complaint during the study period was calculated. Patients presenting with pain were also evaluated according to age and sex. For age-stratified analyses, patients were categorized into three predefined age groups: 3–4 years, 5–9 years, and 10–18 years.

Pain was classified according to duration. Pain lasting less than 3 months was classified as acute, whereas pain lasting 3 months or longer was classified as chronic, in accordance with the definition used in this study (6). The distribution of acute and chronic pain was assessed, and pain duration was additionally evaluated according to age and sex.

Information on age, sex, pain duration, pain location, accompanying systemic findings, diagnoses established after clinical evaluation, consultations requested during the outpatient visit, and diagnostic investigations ordered by the treating physician was extracted from the hospital information system. Laboratory and other diagnostic test results were also reviewed through the same system. Prescription records were reviewed through the electronic prescription system to determine whether an analgesic had been prescribed.

No standardized pain-intensity or multidimensional pain-assessment scale was routinely used in the outpatient records; therefore, pain assessment in this study was based on the complaints and clinical information documented in the medical records.

### 2.4 Follow-up of patients with chronic pain

Patients classified as having chronic pain were followed through the hospital information system to determine whether they attended subsequent clinical visits. Patients who did not return for follow-up were contacted approximately 3 months after the initial presentation using the contact information available in the hospital information system and were asked whether their pain had persisted, improved, or resolved.

No additional diagnostic tests, clinical interventions, or medications were administered specifically for the purposes of the study.

The study flow, including patient screening, identification of pain-related presentations, and classification according to pain duration, is summarized in Figure 1.

**Figure 1:**
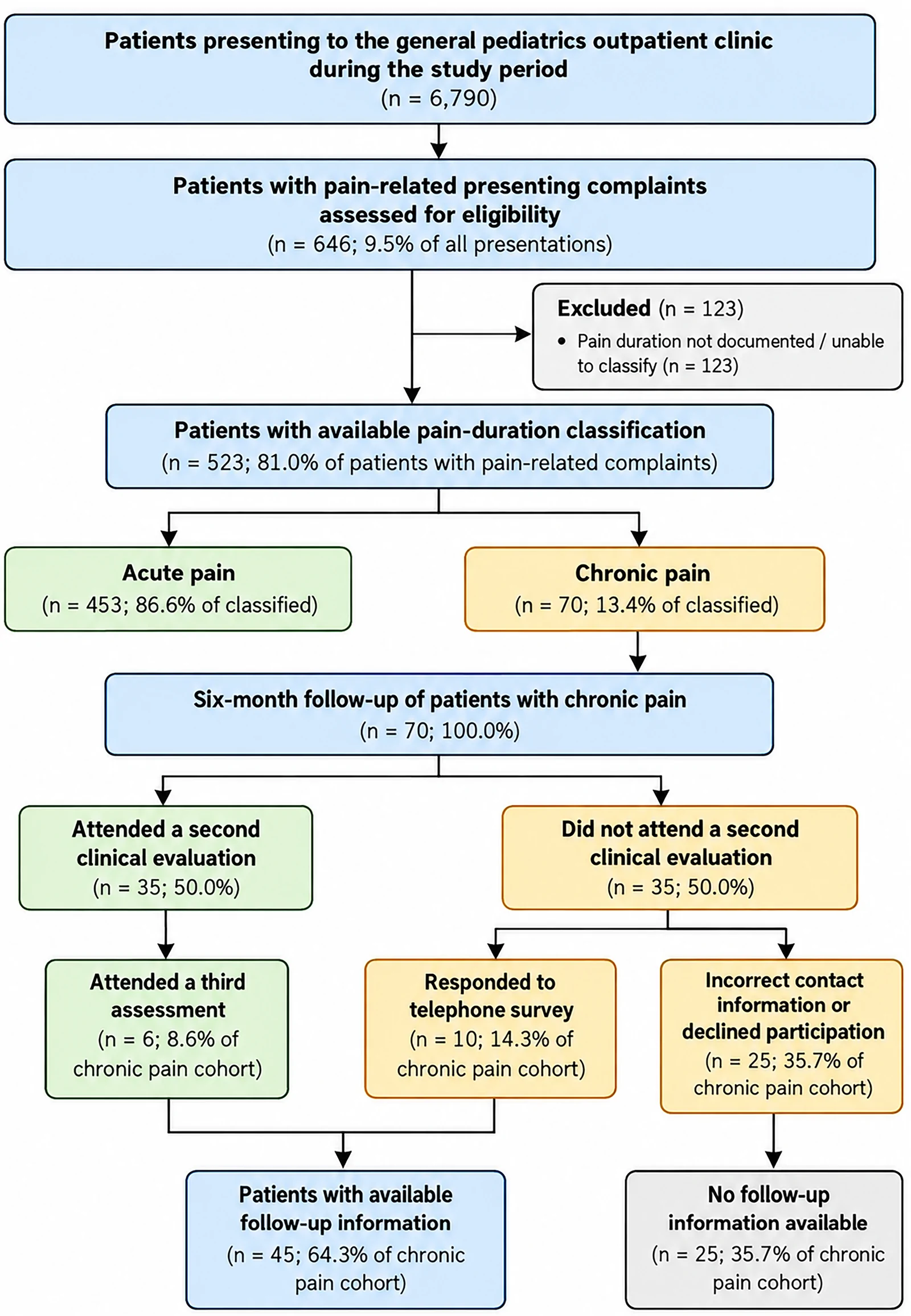
Flow diagram of patient selection, pain classification, and six-month follow-up of patients with chronic pain.

### 2.5 Measures to reduce bias

To reduce selection bias, all eligible outpatient visits occurring during the predefined study period were systematically screened using the same eligibility criteria. Clinical variables were extracted from routinely recorded medical documentation, and analgesic prescribing was evaluated using the electronic prescription system rather than patient recall. The same predefined criteria were used to classify pain duration and age groups throughout the analysis.

### 2.6 Statistical analysis

Statistical analyses were performed using IBM SPSS Statistics version 22.0 (IBM Corp., Armonk, NY, USA). Continuous variables were summarized using means and standard deviations and minimum–maximum values, as appropriate. Categorical variables were presented as frequencies and percentages. Categorical variables were compared using Pearson's chi-square test, Yates’ continuity-corrected chi-square test, or Fisher's exact test, as appropriate based on expected cell frequencies. All statistical tests were two-sided, and a *p* value < 0.05 was considered statistically significant.

To identify independent predictors of chronic pain, multivariable binary logistic regression analysis was performed. Patients with undocumented pain duration (*n* = 123) were excluded from this analysis. Chronic pain (yes/no) was entered as the dependent variable, whereas age, sex and pain location were included as independent variables. A second multivariable binary logistic regression model was constructed to identify factors independently associated with analgesic prescribing. Analgesic prescription (yes/no) was used as the dependent variable. Physical examination findings (normal/abnormal), age, sex, and chronic pain status were included as independent variables. Results of the multivariable logistic regression analyses were reported as adjusted odds ratios (aORs) with 95% confidence intervals (95% CIs).

### 2.7 Ethical considerations

The study was conducted in accordance with the principles of the Declaration of Helsinki. Ethical approval was obtained from the Sami Ulus Children's Hospital Clinical Research Ethics Committee (decision no. 2020-KAEK-141/405). No additional diagnostic procedures or treatments were performed solely for research purposes.

## 3 Results

### 3.1 Demographics

In February 2023, a total of 6,790 patients aged 3–18 years presented to the general pediatric outpatient clinic. Of these, 646 patients (9.5%) presented with pain-related complaints. Table 1 summarizes the demographic characteristics of these 646 patients.

**Table 1:** Demographic characteristics of patients with pain-related complaints.

Sex (female/male) (n, %)	346/300 (53.6/46.4)
Age distrubition (n, %)	
3–5 years	112 (17.3)
5–10 years	240 (37.2)
10–18 years	294 (45.5)
Age (mean ± SD)	9.8 ± 4.5

The proportion of female patients was significantly higher in the adolescent group (58.4% vs. 41.6%, *p* = 0.007), whereas the younger age groups had similar sex distributions.

### 3.2 Types of pain-related complaints

The most common pain-related complaint was sore throat (*n* = 183, 28.3%), followed by abdominal pain (*n* = 147, 22.8%) and headache (*n* = 86, 13.3%). The detailed distribution is presented in Table 2.

**Table 2:** Distribution of pain-related complaints among patients (*n* = 646).

Complaint	Number of patients	Percentage (%)
Sore throat	183	28.3
Abdominal pain	147	22.8
Headache	86	13.3
Chest pain	79	12.2
Ear pain	58	9.0
Extremity pain	39	6.1
Joint pain	20	3.1
Low back and back pain	20	3.1
Other	14	2.1
Total	646	100

### 3.3 Clinical evaluation

Physical examination: No abnormalities were identified on physical examination in 360 patients (55.7%). The most common abnormal finding was oropharyngeal hyperemia and hypertrophy consistent with an upper respiratory tract infection, observed in 210 patients (32.5%).

Diagnostic evaluation: Following the initial evaluation, 376 patients (58.2%) underwent diagnostic testing, including laboratory tests, radiological examinations, throat cultures, and/or urinalysis. Abnormal findings were detected in 231 patients, whereas 145 patients (38.5% of those tested) had normal results. The most frequent abnormalities were nutritional deficiencies, including vitamin D, vitamin B12, and iron deficiency (*n* = 162, 70.1% of abnormal findings).

Final diagnoses: Infections were the most common diagnoses (*n* = 335, 51.8%), mainly upper respiratory tract infections (*n* = 214, 33.1%). Musculoskeletal causes (*n* = 129, 19.9%) and gastrointestinal disorders (*n* = 72, 11.1%) were also common. Despite diagnostic evaluation, the etiology of pain remained unexplained in 44 patients (6.8%).

Among patients presenting with a pain-related complaint, six (0.9%) revisited the outpatient clinic within 1 week with the same complaint.

### 3.4 Hospitalization and referral

Although most cases were benign and self-limiting, a small subset involved potentially serious conditions, including acute abdomen, arthritis, Henoch–Schönlein purpura (HSP), acute rheumatic fever (ARF), nephrolithiasis, and acute renal failure, which may be associated with substantial morbidity and, rarely, mortality.

Six patients required hospitalization. Of these, two were admitted to the pediatric surgery service, one to the pediatric ward with severe gastritis, one with acute gastroenteritis, and two because of inability to tolerate oral intake accompanied by dehydration. All patients were discharged in good clinical condition, with no ongoing life-threatening condition identified.

A total of 153 patients (23.7%) were referred to subspecialty clinics for further evaluation and follow-up, most commonly to neurology. One patient with headache was referred to neurosurgery after cranial MRI revealed a 4.5-mm microaneurysm in the left posterior cerebral artery (PCA), and another was referred to neurology with a preliminary diagnosis of epilepsy because of transient visual blurring and jaw clenching.

### 3.5 Management

Among the patients, 229 (35.5%) received antibiotics, 168 (26.0%) analgesics, and 118 (18.3%) vitamin supplements, whereas no medication was prescribed for 192 patients (29.7%). Paracetamol and ibuprofen were the only analgesics prescribed; no opioids were prescribed. Fifty-one patients (7.9%) received symptomatic treatment for abdominal pain or constipation, including lactulose, a proton pump inhibitor, an enema, or trimebutine maleate. Of the 44 patients with unexplained pain, 40 received no analgesic therapy.

The proportion of patients receiving analgesic treatment was significantly higher among those with acute pain than among those with chronic pain (30.7% vs. 11.4%, *p* = 0.001). Analgesic treatment was prescribed significantly more frequently to patients with abnormal physical examination findings than to those with normal findings (44.4% vs. 14.2%, *p* < 0.001). Analgesic treatment was prescribed significantly more frequently to male than to female patients (31.7% vs. 24.0%, *p* = 0.029).

Analgesic prescribing differed significantly across age groups, with the highest rate observed among children aged 3–4 years (38.4%), compared with 23.8% among those aged 5–9 years and 26.5% among those aged 10–18 years (*p* = 0.014). Figure 2 presents analgesic prescription rates across age groups, stratified by sex.

**Figure 2:**
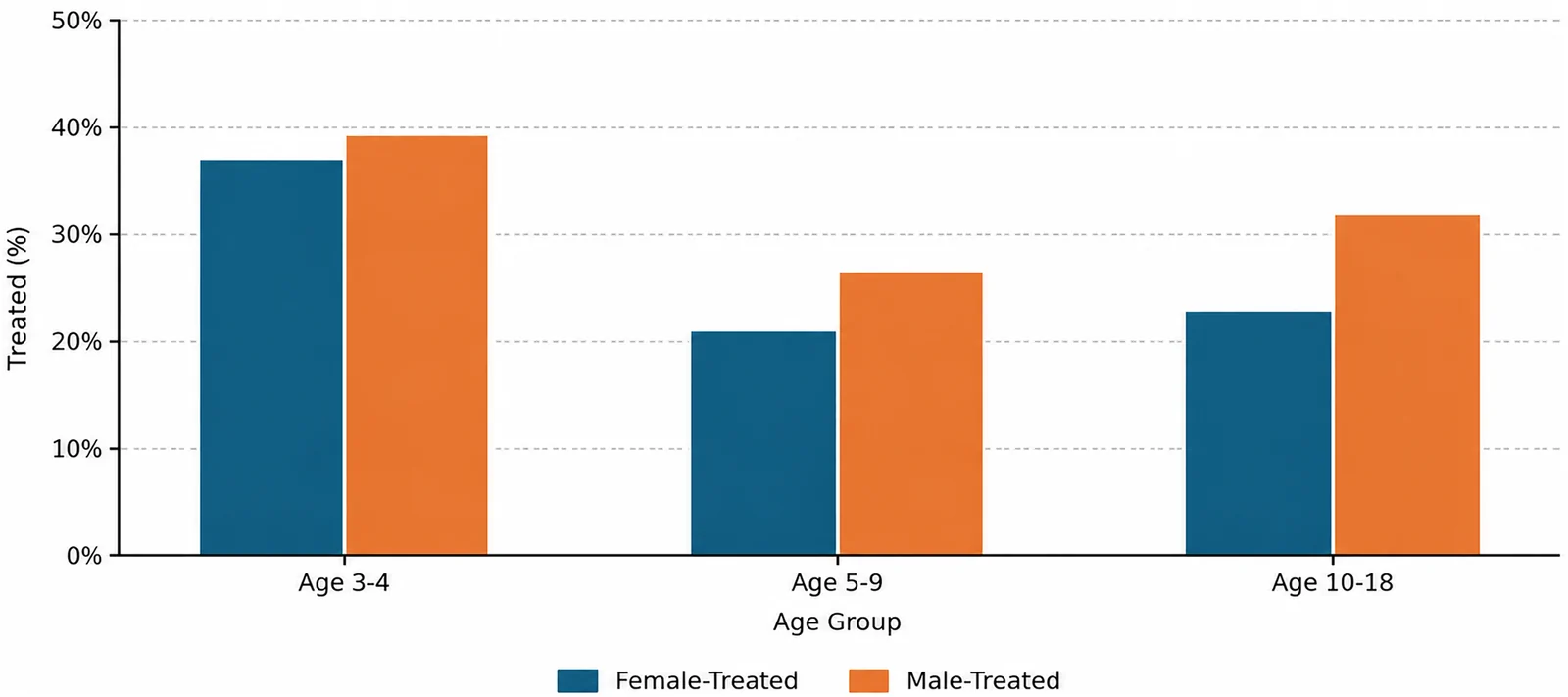
Analgesic Prescription Rates According to Age Group and Sex.

Variables significantly associated with analgesic treatment in univariable chi-square analyses (age, sex, chronic pain status, and abnormal physical examination findings) were included in the multivariable logistic regression model. The overall model was statistically significant. After adjustment, age (*p* = 0.156), sex (*p* = 0.124), and chronic pain status (*p* = 0.182) were no longer significantly associated with analgesic treatment. Abnormal physical examination findings remained the only independent predictor of analgesic prescribing (aOR 5.10, 95% CI 3.25–7.98; *p* < 0.001), indicating approximately fivefold higher odds of receiving an analgesic among patients with abnormal examination findings.

### 3.6 Pain duration

Analysis of pain duration showed that 453 patients (70.1%) presented with acute pain and 70 (10.8%) with chronic pain, whereas pain duration was not documented in 123 patients (19.0%). Chronic pain was more frequent among adolescents (10–18 years: *n* = 45, 64.3%) than among younger children (3–4 years: *n* = 7, 10.0%; 5–9 years: *n* = 18, 25.7%), while acute pain predominated in all age groups (*p* = 0.001). In multivariable logistic regression analysis, each one-year increase in age was associated with an 11% increase in the odds of chronic pain (aOR 1.11, 95% CI 1.05–1.18). Chronic pain was more prevalent among female than male patients (68.6% vs. 31.4%, *p* = 0.007), and female sex was independently associated with 1.85-fold higher odds of chronic pain (aOR 1.85, 95% CI 1.07–3.19). Among patients with chronic pain, headache was the most frequent presenting complaint (*n* = 29, 41.4%); other complaints are summarized in Table 3. Pain location was not independently associated with chronic pain (*p* = 0.214).

**Table 3:** Presenting complaints among patients with chronic pain.

Complaint	Number of patients	Percentage (%)
Headache	29	41.4
Abdominal pain	21	30.0
Extremity pain	13	18.5
Chest pain	2	2.9
Joint pain	2	2.9
Back pain	2	2.9
Flank pain	1	1.4
Total	70	100

At the initial visit, definitive diagnoses were established in 14 patients with chronic pain (Table 4). Patients without a conclusive diagnosis at the first visit were referred to appropriate subspecialty clinics with preliminary working diagnoses. The most common preliminary diagnoses were primary headache (*n* = 22) and functional abdominal pain (*n* = 10) (Table 4).

**Table 4:** Diagnostic evaluation of patients with chronic pain at the initial visit.

Diagnosis	Number of patients	Percentage (%)
No diagnosis	10	14.3
Definitive diagnoses	14	20
• Myalgia	6	8.5
• Sinusitis	2	2.8
• Endometrioma	1	1.4
• Dysmenorrhea	1	1.4
• C. difficile infection	1	1.4
• Nephrolithiasis	1	1.4
• Serositis	1	1.4
• Mesenteric lymhadenitis	1	1.4
Preliminary diagnoses with subspecialty referral	46	65.7
• Primary Headache	22	31.4
• Functional Abdominal Pain	10	14.2
• Mechanical Joint Pain	7	10
• Growing pain	2	2.8
• Gastritis	2	2.8
• Gastroesophageal Reflux Disease	2	2.8
• Constipation	1	1.4
Total	70	100

During the 6-month follow-up period, 35 of 70 patients (50.0%) with chronic pain attended a second clinical evaluation, and six patients (8.6%) underwent a third assessment. Among the 35 patients who did not attend a second evaluation, 10 responded to the telephone survey, whereas 25 had incorrect contact information or declined participation. Thus, follow-up information was available for 45 patients. Of these, 20 (44.4%) reported persistent pain, 11 (24.4%) reported improvement, and 14 (31.1%) reported complete resolution. The six-month follow-up outcomes of patients with chronic pain are summarized in Figure 3.

**Figure 3:**
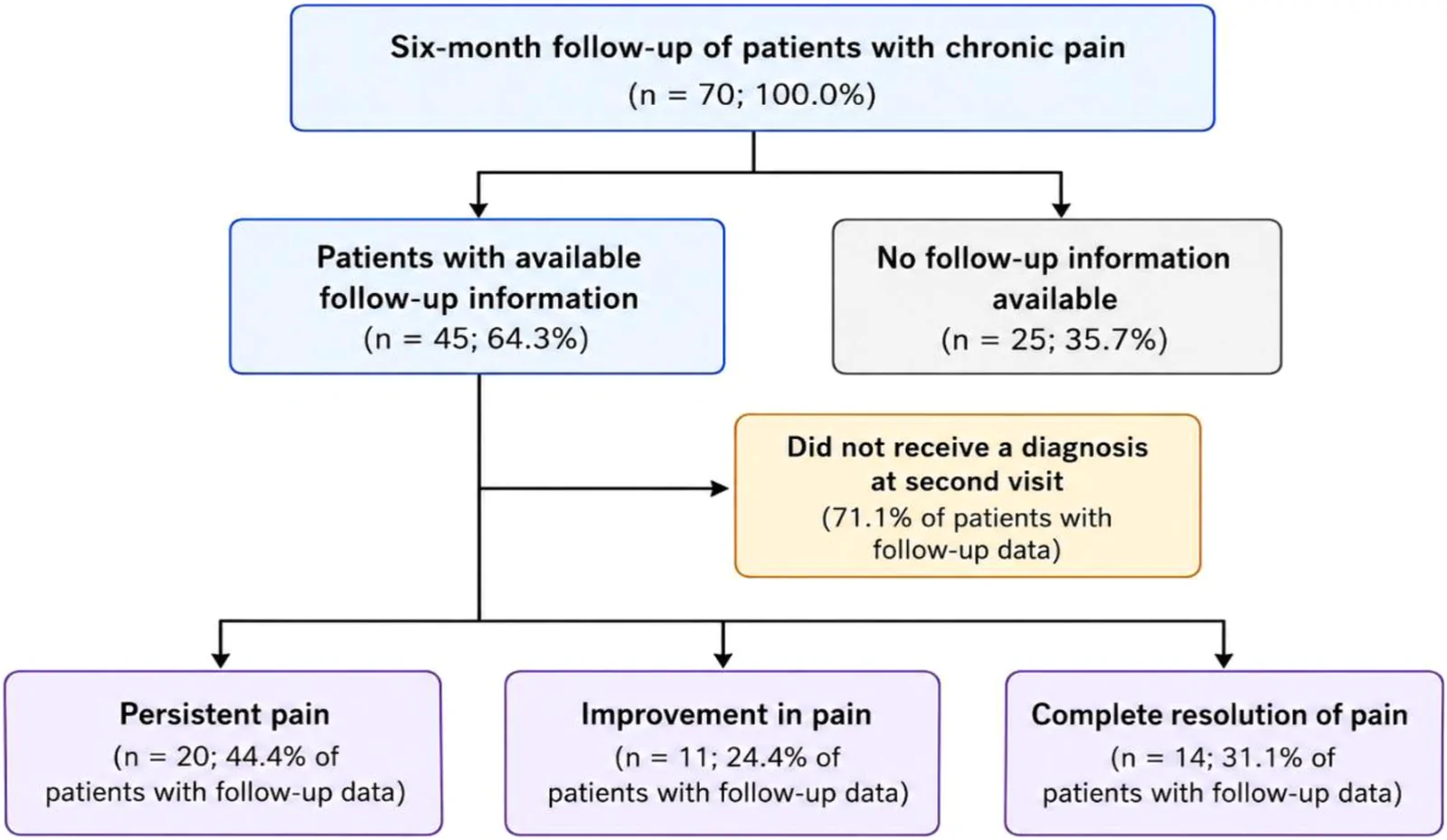
Six-month follow-up outcomes of patients with chronic pain.

At follow-up, 32 of 45 patients (71.1%) had no documented definitive diagnosis. Diagnoses identified during follow-up are presented in Table 5.

**Table 5:** Diagnostic status of patients with chronic pain at follow-up.

Diagnosis	Number of patients	Percentage (%)
No diagnosis	32	71.1
Migraine	4	8.8
Myalgia/Fibromyalgia	3	6.6
Constipation	2	4.4
Chronic gastritis	2	4.4
Dysmenorrhea	1	2.2
Gastroesophageal reflux	1	2.2
Total	45	100

At the third assessment, three patients reported resolution of pain, two reported persistent pain, and one reported worsening symptoms (*n* = 6). The patient with worsening pain had previously been diagnosed with migraine and had started beta-blocker therapy; because of newly developed allergic symptoms, a pediatric allergy consultation was requested. Two other patients continued to experience pain without a definitive diagnosis.

Among patients with chronic pain, only two were prescribed analgesic medication. Vitamin supplementation was prescribed to 30 patients, while three received gastrointestinal-specific pharmacological therapy, including proton pump inhibitors and antacids.

## 4 Discussion

Pain is a common and increasingly recognized health problem among children and adolescents. In the present study, 9.5% of patients presenting to the general pediatric outpatient clinic reported pain as their presenting complaint. Previous studies evaluating pain prevalence in pediatric populations have used different methodological approaches, including questionnaire-based surveys and retrospective database analyses. In a school-based questionnaire study conducted in the Lübeck region of Germany, Roth-Isigkeit et al. reported that 85.3% of 735 children and adolescents aged 10–18 years had experienced pain within the previous 3 months (7). Similarly, in a school-based survey conducted in the Rotterdam area, Perquin et al. found that 53.7% of children aged 0–18 years reported pain during the preceding 3 months (8). In contrast, studies conducted in clinical settings have reported lower rates. Grout et al., in a cross-sectional study of 26,180 patients aged 2–19 years in pediatric primary care, reported pain in 14.9% of visits and found that pain reporting was associated with obesity, adolescence, female sex, and previous pain history (9). The lower proportion observed in our study may be related to the retrospective component of the design, the inclusion of only pain as a presenting complaint, and the possibility that pain was underdocumented in routine outpatient records.

Previous studies have consistently shown that girls report pain more frequently than boys. In the questionnaire study by Perquin et al., 57.3% of patients reporting pain were female and 42.7% were male. The same study also showed that 60.1% of girls and 46.9% of boys had experienced pain within the previous 3 months (8). Similarly, Haraldstad et al. reported pain in 65% of girls and 55% of boys in a Norwegian survey (1). Although sex-related differences in pain have received increasing attention, much of the existing evidence has focused on adult populations, and fewer studies have specifically examined these differences in pediatric pain (10).

Consistent with the literature, our findings showed that pain-related complaints were more frequent among girls and increased with age, particularly during adolescence. This pattern may reflect multiple biological, psychological, and social factors. Hormonal changes during puberty, psychosocial stress, emotional vulnerability, and differences in pain perception and reporting may contribute to the higher frequency of pain among adolescent girls (10). In addition, older children and adolescents may be better able to recognize, localize, and verbalize pain, which may partly explain the age-related increase observed in our study.

The most common pain-related reason for presentation in our cohort was sore throat, followed by abdominal pain and headache. The predominance of sore throat may be related to seasonal upper respiratory tract infections, which are among the most frequent reasons for pediatric outpatient visits. Among patients presenting with headache, the absence of an identifiable organic cause in a substantial proportion of cases may reflect contributions from lifestyle-related factors, including prolonged screen exposure, irregular sleep patterns, academic stress, and psychosocial factors. Among patients with abdominal pain, benign and functional causes appeared to predominate.

Although acute pain presentations were more common overall, chronic pain was identified in 10.8% of patients, with higher rates particularly among adolescent girls. Haraldstad et al. reported that 21% of children and adolescents experienced chronic pain. In that study, no statistically significant sex-based difference in pain duration was observed; however, the frequency of chronic pain increased with age in both sexes (1). In a 2024 meta-analysis, Chambers et al. reported an overall prevalence of chronic pain of 20.8% among children and adolescents, with the highest prevalence for headache and musculoskeletal pain (6). In our study, headache was also the most common presenting complaint among patients with chronic pain.

Our findings suggest potential gaps in pain assessment and management in the outpatient setting. Pain duration was not documented in a considerable proportion of patients, and only 26% received analgesic treatment. Moreover, most patients with unexplained pain did not receive analgesic therapy, which may indicate gaps in symptomatic pain management. Opioid use may represent an additional parameter when evaluating pain management practices, particularly in patients with moderate to severe pain. Because opioid analgesics are controlled medications subject to strict regulatory oversight, prescribing may be influenced not only by clinical indications but also by physicians’ knowledge, attitudes, concerns, and confidence in pain management (11). Opioid consumption has also been proposed as an indirect indicator of access to adequate pain treatment at the population level (12). However, pain severity was not assessed in our study, and the prescribed analgesics were limited to paracetamol and ibuprofen. Therefore, opioid use could not be evaluated as a marker of pain-management adequacy. Further studies should assess pediatric outpatient pain management more comprehensively, including pain severity, analgesic class, treatment appropriateness, and clinical response.

Our multivariable analysis provided further insight into factors associated with analgesic prescribing. Although younger age, male sex, acute pain, and abnormal physical examination findings were associated with analgesic treatment in univariable analyses, only abnormal physical examination findings remained independently associated with analgesic prescribing after adjustment for age, sex, and pain chronicity (aOR 5.10, 95% CI 3.25–7.98; *p* < 0.001). This finding suggests that clinicians may be more likely to initiate analgesic treatment when pain is accompanied by an objective physical finding, whereas age, sex, and pain chronicity were not independently associated with prescribing after adjustment. The strong association with abnormal examination findings may indicate that treatment decisions are influenced by clinically observable pathology. The higher analgesic prescribing rate observed in younger children may partly reflect the dual analgesic and antipyretic effects of commonly used agents such as paracetamol and ibuprofen, which may also have been prescribed for concomitant fever. Similarly, the lower prescribing rate among patients with chronic pain may reflect a greater emphasis on investigating the underlying cause of persistent symptoms rather than providing immediate symptomatic treatment.

Baldridge et al., in their study of outpatient pain treatment in pediatric patients, similarly reported that a substantial proportion of children with pain-related diagnoses did not receive prescribed pain treatment (3). In a cross-sectional study published in the Italian Journal of Pediatrics, pain prevalence, pain intensity, and the adequacy of analgesic treatment were evaluated among hospitalized children and adolescents in pediatric surgery and onco-hematology units at Ospedale Pediatrico Bambino Gesù. Using the Pain Management Index, the authors found that 87% of patients with moderate to severe pain who received analgesic treatment had a PMI score below zero, indicating inadequate pain management in this group (13). This finding emphasizes that effective pain control requires not only treatment initiation but also treatment selection appropriate to pain severity. Healthcare professionals play a central role in pediatric pain management. In a 2023 survey of Italian anesthesiologists’ perceived self-efficacy and educational needs regarding pain management, the lowest level of perceived competence was reported for pediatric pain management (14). This finding highlights the need to strengthen professional education and training in pediatric pain assessment and treatment. In addition, time constraints, staff shortages, heavy workload, lack of standardized protocols, and insufficient institutional prioritization of pain management have been identified as barriers to effective pain control in pediatric patients (15). More structured, patient-centered, and standardized pain-management strategies are therefore needed in pediatric outpatient practice.

Another noteworthy finding was that physical examination findings were mostly normal and that a substantial proportion of diagnostic investigations did not reveal a specific pathology. Grout et al. similarly reported that 90% of children presenting with pain in primary care had no abnormal physical examination findings (9). The relatively high frequency of diagnostic testing in our cohort, together with the substantial proportion of investigations that did not identify a specific etiology, may reflect diagnostic uncertainty in children presenting with pain. Similar findings have been reported in children with nonspecific or functional abdominal pain, in whom routine laboratory and imaging investigations have demonstrated limited diagnostic yield in the absence of alarm features. Wallis and Fiks reported that 70% of children with nonspecific abdominal pain remained without a specific diagnosis during follow-up and that the diagnostic yield of laboratory investigations was approximately 3% (16). Similarly, Llanos-Chea and Saps emphasized a selective diagnostic approach guided by clinical assessment and alarm features (17). Evidence from pediatric headache populations is consistent with this approach; Lewis and Dorbad found limited clinical utility of routine neuroimaging in children with uncomplicated migraine or chronic daily headache and normal neurological examinations because identified abnormalities did not alter management or outcomes (18). Collectively, these findings support a selective diagnostic strategy based on clinical red flags and examination findings rather than routine testing.

A strength of our study is the inclusion of a broad pediatric age range (3–18 years) and multiple types of pain in a general pediatric outpatient setting, providing a comprehensive perspective on pain-related presentations in routine practice. The inclusion of both acute and chronic pain also allows a broader characterization of the clinical spectrum of pediatric pain.

The primary limitations of this study relate to its single-center design and the retrospective component based on electronic health records. Because the analysis depended on routine clinical documentation, incomplete or inconsistent records may have resulted in underascertainment of pain-related presentations, pain duration, and analgesic prescribing, thereby introducing information bias. In addition, the absence of standardized pain-assessment scores precluded evaluation of pain severity, functional impairment, and treatment response. The retrospective component also limited access to potentially relevant clinical and psychosocial variables and precluded causal inference regarding associations between patient characteristics and pain-management practices. Furthermore, because the study was conducted at a single tertiary pediatric outpatient center and data collection was restricted to 1 month, the findings may be affected by seasonal variation and may not be fully generalizable to primary care or other healthcare settings.

In conclusion, pain is a common reason for presentation in pediatric outpatient practice and is more frequently reported among girls and adolescents. Although acute pain was more common, chronic pain was also identified in a notable proportion of patients, particularly among adolescent girls. The low rate of analgesic prescribing and incomplete documentation of pain characteristics indicate important gaps in outpatient pediatric pain management. Implementation of standardized pediatric pain-assessment tools, structured documentation protocols, and targeted physician education may support more consistent, evidence-based, and patient-centered pain care for children and adolescents.

## Data Availability

The original contributions presented in the study are included in the article/supplementary material, further inquiries can be directed to the corresponding authors.
